# Mid-ileal Volvulus Treated With Urgent Small Bowel Resection: A Case Report

**DOI:** 10.7759/cureus.81042

**Published:** 2025-03-23

**Authors:** Mohammad Kloub, Abdul-Rahman I Abusalim, Abdelhadi Farouji, Mohamed Eldesouki, Atheer Anwar, Muhammad Hussain, Yatinder Bains

**Affiliations:** 1 Department of Internal Medicine, New York Medical College, Saint Michael's Medical Center, Newark, USA; 2 Department of Internal Medicine, University of Wisconsin School of Medicine and Public Health, Madison, USA; 3 School of Medicine, Mutah University, Karak, JOR; 4 Department of Gastroenterology and Hepatology, New York Medical College, Saint Michael's Medical Center, Newark, USA; 5 Department of Gastroenterology, New York Medical College, Saint Michael's Medical Center, Newark, USA

**Keywords:** intestinal necrosis, intestinal obstruction, primary small bowel volvulus, small bowel ischemia, surgical resection

## Abstract

Volvulus is a rare condition characterized by intestinal twisting that requires immediate medical intervention due to its potentially life-threatening complications such as bowel obstruction, ischemia, and perforation. The majority of volvulus cases occur in the colon, while those in the small intestine are exceedingly rare. We present the case of a 77-year-old patient with a complex medical history who presented with abdominal pain and signs of intestinal obstruction due to an ileal volvulus. This case highlights the importance of considering this unusual entity in the differential diagnosis of acute abdominal discomfort.

## Introduction

Volvulus is a medical term that originated from the Latin word volvere, which means "to twist." It was first described in the literature by von Rokitansky in 1841 [1]. Twisting of the intestine is the hallmark of intestinal volvulus, which most commonly affects the sigmoid colon. This pathology is known for its potential to cause obstruction, ischemia, and intestinal necrosis, with serious consequences [2]. While volvulus most commonly occurs in the sigmoid colon, it can occur throughout the gastrointestinal tract [2].

Colonic volvulus is the third most common cause of large bowel obstruction globally. It predominantly affects populations in Africa, the Middle East, India, and Russia [3]. Small bowel volvulus (SBV), which involves the twisting of a portion of the small intestine and its mesentery, is a rare and potentially life-threatening cause of gastrointestinal obstruction. It accounts for 1-4% of cases in Western countries, but this figure rises to 20-35% in regions such as Asia, Africa, and the Middle East. This is mainly attributed to genetic, anatomical, and dietary factors increasing the risk in these regions [3,4].

The treatment of colonic and SBV depends on factors such as the location, underlying cause, and any complications present at the time of diagnosis. It may involve both surgical and non-surgical approaches [2].

In this case report, we describe a 77-year-old woman who had a small bowel resection after presenting with abdominal pain and being diagnosed with an ileal volvulus, and we also provide a brief discussion on this rare clinical entity.

This article was previously posted to the Authorea preprint server on June 03, 2024.

## Case presentation

A 77-year-old female patient with a medical history of well-controlled chronic obstructive pulmonary disease (COPD) and breast cancer in remission status post-lumpectomy presented with generalized abdominal pain of acute onset that persisted for one week prior to her presentation. The pain was intermittent, non-radiating, rated 8/10, and accompanied by bright red blood per rectum that has turned black for the last two days. There were no accompanying symptoms of vomiting, constipation, or diarrhea. She denied any fever, chills, chest pain, palpitations, or changes in urinary habits.

The patient had a previous hysterectomy 10 years ago and removal of a benign sigmoid polyp that was detected on a routine colonoscopy one year prior to presentation.

Upon physical examination, the patient appeared to be in pain and mild distress. Vital signs showed the following: heart rate (HR): 102, blood pressure (BP): 128/81, temperature: 37.1°C, and oxygen saturation (SpO2): 96%. She had generalized abdominal tenderness without guarding or rebound and no palpable masses. The cardiac exam revealed tachycardia (HR: 102) with a regular rhythm, normal S1 and S2, and no added sounds. The chest exam showed normal air entry bilaterally with no added sounds. The neurological exam was non-focal.

Initial blood tests, including complete blood count (CBC), comprehensive metabolic panel (CMP), and lactic acid, were within normal limits (Table 1).

**Table 1:** Initial blood tests on presentation including CBC, CMP, lipase, INR, and lactic acid. WBC: white blood cell; BUN: blood urea nitrogen; ALT: alanine aminotransferase; AST: aspartate aminotransferase; ALP: alkaline phosphatase; INR: international normalized ratio; CBC: complete blood count; CMP: comprehensive metabolic panel

Result	Value	Reference range
Hemoglobin	12.2 g/dL	12-15.5 g/dL
WBC	8.5x10^3^/μL	4.5-11.0x10^3^/μL
Platelets	410x10^3^/μL	150-450x10^3^/μL
Creatinine	0.91 mg/dL	0.5-1.0 mg/dL
BUN	16 mg/dL	8-20 mg/dL
ALT	30 U/L	4-36 U/L
AST	34 U/L	10-40 U/L
ALP	56 IU/L	44-147 IU/L
Bilirubin, total	0.3 mg/dL	0.1-1.2 mg/dL
Lipase	40 U/L	13-78 U/L
Lactic acid	1.2 mg/dL	0.4-2.0 mg/dL
INR	1.0	<1.2

**Figure 1:**
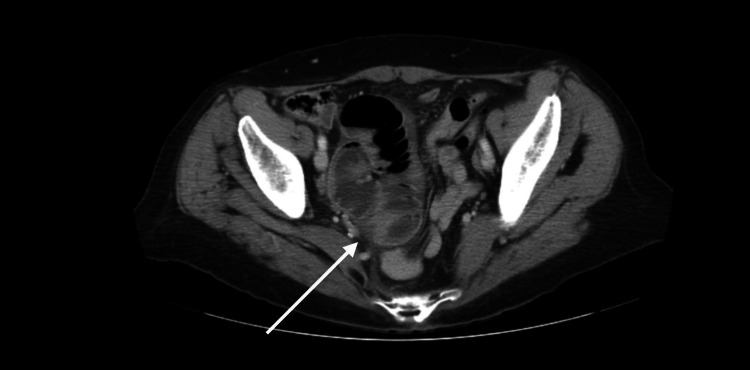
CT of the abdomen shows prominent mid-ileal loops with torsion of ileal loops around its mesentery, suggestive of probable mid-ileal volvulus (white arrow). CT: computed tomography

Computed tomography (CT) scan of the abdomen with intravenous (IV) contrast revealed prominent mid-ileal loops with torsion of ileal loops around its mesentery, suggestive of probable mid-ileal volvulus as shown in Figure 1. 

The patient was admitted to the hospital, a nasogastric tube was placed for decompression, she was kept nothing by mouth (NPO) and started on IV fluids, and analgesia was provided.

The surgical team was consulted, and an exploratory laparotomy was performed. It revealed malrotation of the small bowel with volvulus and obstruction, along with significant adhesions overlying the small bowel at a different segment than the twisted bowel. There were no signs of necrosis or ischemic bowel; however, due to the redundancy of a large segment of the bowel, lysis of adhesions and small bowel resection from the duodenojejunal junction to the ileocecal valve were performed. 

Post-operatively, the patient was started on a clear liquid diet. She had one day of constipation post-operatively, which resolved by post-operative day 2. By day 3, she was able to pass bowel movements and tolerated a full diet. The surgical scar was healing appropriately, with no surgical complications identified.

She was discharged in a stable condition with complete resolution of her symptoms. Outpatient surgical and nutrition follow-up was provided for post-operative evaluation. 

## Discussion

Colonic volvulus is considered the third leading cause of large bowel obstruction worldwide, responsible for around 15% of large bowel obstructions in the United States [2]. It most commonly affects the sigmoid colon, representing about 60% of cases, followed by the cecum, which represents 34% of all cases [5]. While volvulus most commonly occurs in the colon, it can affect any part of the gastrointestinal tract, including the stomach and small bowel. SBV is considered a rare cause of small bowel obstruction, representing 1-4% of cases in the Western world [4,6]. 

The clinical manifestations of volvulus are nonspecific and can be varied depending on the location of the volvulus. Patients with sigmoid volvulus typically present with abdominal distention (79%), followed by pain (58%) and obstipation (55%), whereas most patients with cecal volvulus present complaining of abdominal pain [7]. On the other hand, patients with SBV may present either acutely in 89% of the cases due to acute vascular insufficiency or peritonitis or with vague symptoms and signs common to other causes of gastrointestinal obstruction (abdominal pain, nausea, vomiting, and distention) [6]. While colonic volvulus affects the mobile parts of the colon, such as the cecum, transverse colon, and sigmoid, SBV has a different pathophysiology when small bowel torsion during volvulus happens around its mesenteric vascular pedicle [2,4].

SBV has been classified into primary and secondary categories based on the cause implemented [6]. Primary SBV has no identifiable underlying predisposing factors and mainly occurs in children and young adults [6]. Secondary SBV mainly arises from an identifiable lesion such as post-operative adhesions or underlying intestinal abnormalities like congenital malrotation, tumors, aneurysms, pregnancy, and diverticulosis. It usually affects those over 40 years of age and is more prevalent in Western nations [6]. 

Diagnosing SBV requires a high index of suspicion and familiarity with its imaging features, as it is a rare condition with diverse clinical presentations and variable signs and symptoms. Early recognition is crucial to prevent serious complications. Plain abdominal radiography can show air-fluid levels in case of intestinal obstruction; when ischemia or necrosis occurs, pneumoperitoneum and pneumatosis intestinalis can be seen. Abdominal CT with IV contrast is considered the most relevant and recommended diagnostic imaging modality [4,6]. 

Treatment options for sigmoid volvulus include non-surgical intervention, including endoscopic detorsion, if there is no concern for complications such as ischemia or gangrenous bowel. Nevertheless, due to the high recurrence rate (approaching 90%), patients should undergo surgical resection afterward [2]. Surgery must be performed immediately if the endoscopic detorsion fails or if there are signs of intestinal necrosis or ischemia [2]. SBV carries a very high risk for bowel ischemia, so surgical intervention is the first choice of treatment to restore the blood perfusion and treat underlying causes including mass or lesion [6,7]. 

## Conclusions

This report highlights the rare and serious nature of SBV due to its high risk of complications, including intestinal obstruction, ischemia, and necrosis if not promptly diagnosed and treated. Given that it is uncommon in Western countries and the diverse and nonspecific nature of its clinical manifestations, it can be difficult to identify without a high index of suspicion and appropriate imaging.

We presented the case of a 77-year-old woman with acute abdominal pain and symptoms suggestive of a mid-ileal volvulus. Our immediate recognition of the signs of SBV and urgent surgical intervention, including lysis of adhesions and small bowel resection, resulted in a full recovery for the patient.

Our report emphasizes the importance of early detection and timely surgical treatment of SBV, especially in cases where there is bowel ischemia or necrosis. Additionally, it reinforces the need for awareness of secondary causes, such as adhesions and malrotation, in the elderly population. Early recognition and prompt intervention remain essential in preventing significant complications and improving patient outcomes.
